# Cooling-induced brushite crystallization in urine as a predictive risk marker for calcium kidney stone recurrence

**DOI:** 10.1007/s00240-025-01820-2

**Published:** 2025-09-06

**Authors:** Yutaro Tanaka, Ichiro Tsujino, Hiroshi Y. Yoshikawa, Kazufumi Takano, Atsushi Okada, Kenjiro Kohri, Takahiro Yasui, Masashi Yoshimura, Yusuke Mori, Mihoko Maruyama

**Affiliations:** 1 Graduate School of Engineering, The University of Osaka, 2-1, Yamadaoka, Suita, 565- 0871 Japan; 2 Department of Nephro-urology, Graduate School of Medical Sciences, Nagoya City University, 1-Kawasumi, Mizuho-cho, Mizuho-Ku, Nagoya, 467-8601 Japan; 3 Graduate School of Life and Environmental Sciences, Kyoto Prefectural University, 1-5, Hangi-cho, Shimogamo, Sakyo-ku, Kyoto, 606-8522 Kyoto Japan; 4 Institute of Laser Engineering, Osaka University, 2-6, Yamadaoka, Suita City, 565- 0871 Osaka Japan

**Keywords:** Kidney stone, Crystalluria, Cooling urine, Calcium oxalate, Brushite, 24 hours urine

## Abstract

Kidney stones have a high recurrence rate—10% within 5 years and 50% within 10. Crystalluria reflects the urinary physicochemical environment and may serve as a recurrence marker, but key crystals like brushite are rarely detected under ambient conditions. This study aimed to identify novel recurrence markers by inducing crystallization through urine cooling and analyzing crystal composition. The analyzed urine samples from 164 stone formers, including first-time stone formers (FSF, *n* = 77) and recurrent stone formers (RSF, *n* = 87). The RSF group was further stratified into low-risk (*n* = 43) and high-risk (*n* = 44) groups based on recurrence intervals. A 24-hour urine test assessed mineral composition and supersaturation indices. Urine samples were then cooled to induce crystallization; precipitated crystals were analyzed using microscopy and Raman spectroscopy. The presence, size, and quantity of brushite crystals were compared among the groups. Before cooling, crystals were detected in only 10.9% of samples, whereas after cooling, crystallization occurred in 76.2%, revealing six crystal types, including calcium oxalate dihydrate (COD) and brushite. COD prevalence did not differ significantly among the groups, whereas brushite crystals were significantly more frequent in the high-risk RSF (47.7%) than in low-risk RSF (16.3%) and FSF (16.9%) (*p* = 0.002) groups. Additionally, the high-risk RSF group had greater quantity and larger size of brushite crystals than did other groups. Cooling-induced brushite crystallization is a promising risk marker for early stone recurrence, with higher crystal quantity and larger size strongly associated with high-risk patients. This method might enhance predictive accuracy beyond traditional 24-hour urine tests, providing a simple, cost-effective tool for recurrence prevention.

**Supplementary Information:**

The online version contains supplementary material available at 10.1007/s00240-025-01820-2.

## Introduction

 Kidney stones are highly recurrent disorders, with recurrence rates reaching approximately 10% within 5 years and up to 50% within 10 years of initial onset [1, 2]. Patients with recurrent stones are at increased risk of complications such as urinary tract infections and progressive renal dysfunction, which may ultimately lead to dialysis or increased mortality [3]. Therefore, early identification of high-risk individuals and implementation of effective preventive strategies are essential. However, current predictive tools—including 24-hour urine analysis and supersaturation index calculations—offer only limited prognostic value for recurrence [4–6].

To understand the mechanisms underlying stone recurrence, we have previously applied mineralogical techniques to examine the internal structure of kidney stones [7, 8]. These stones are primarily composed of calcium oxalate (CaOx) crystals, including monohydrate (COM) and dihydrate (COD) crystals, which aggregate and incorporate urinary proteins during stone development. In addition to CaOx, we have observed the presence of calcium phosphate (CaP) crystals within stones, including not only apatite crystals that contribute to Randall’s plaques (RPs), but also brushite-like CaP crystals that appear to have precipitated directly from urine [9]. Notably, we identified instances where COM or COD crystals were anchored to these CaP crystals, suggesting that diverse urinary crystals—especially brushite—may act as structural triggers in stone pathogenesis.

Crystalluria is clinically useful for the diagnostic evaluation of stone disease and reflects the urinary physicochemical environment [10]. However, crystalluria is detected in fewer than 10% of urine samples under routine testing at ambient temperature [11]. CaOx crystals account for the majority of cases (approximately 80%) but are also commonly observed in healthy individuals, limiting their diagnostic utility [12–14]. In contrast, CaP crystals—particularly brushite—are detected in only 0.8% of samples and have not yet been established as recurrence markers. Nevertheless, brushite stones are clinically associated with high recurrence and resistance to fragmentation, underscoring the need to explore brushite crystallization in more detail.

In this study, we focused on a cooling-induced crystallization technique that promotes crystal nucleation and growth by increasing urinary supersaturation through temperature reduction [15]. We hypothesized that this method could enhance the detection of otherwise rare crystals such as brushite, and that their presence or abundance might be indicative of an elevated risk of recurrence. Thus, our aim was to evaluate cooling-induced crystalluria, with particular emphasis on brushite crystallization, as a novel, noninvasive biomarker for predicting calcium stone recurrence.

## Methods

### Ethics statement

The research project presented in this paper was approved by the Institutional Review Board of the Graduate School of Medicine, Nagoya City University (approval number: 60-24-0034). An opt-out text in accordance with procedures approved by the Ethics Committee Board was posted to research participants, guaranteeing them the opportunity to refuse to participate in the research.

### Participants

**Fig. 1:**
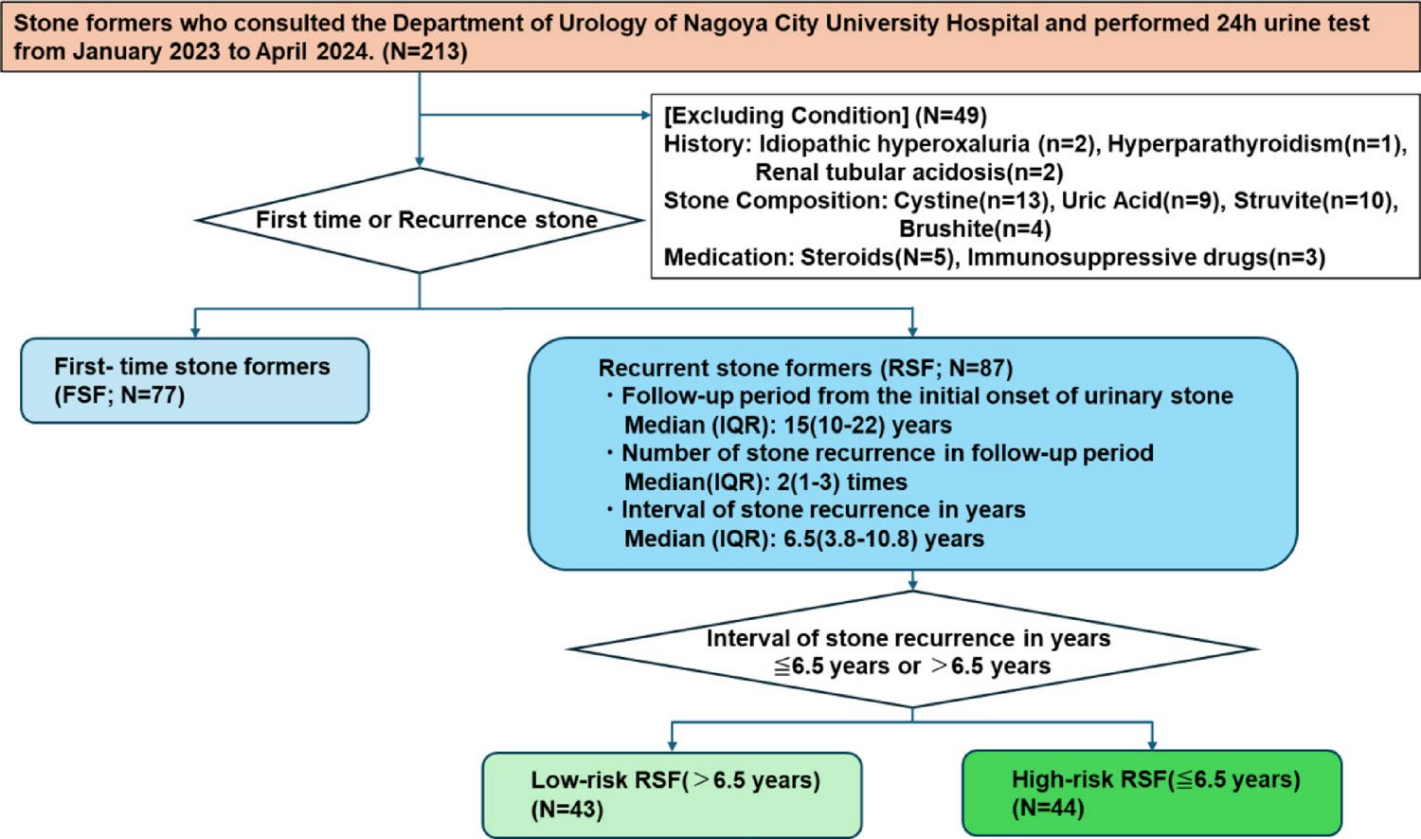
Flowchart diagram of the study design. This flowchart shows how the participants were screened. A total of 213 stone formers who consulted the Department of Urology at Nagoya City University Hospital and underwent 24-hour urine collection tests between January 2023 and April 2024 were included in this study. Excluding conditions based on history, stone composition and medication. Participants were categorized into two groups: first-time stone formers and recurrent stone formers. Furthermore, based on the recurrence interval data, RSF were further stratified into two subgroups: high-risk RSF, defined as those with an interval to recurrence of ≤ 6.5 years, and low-risk RSF, defined as those with an interval to recurrence of > 6.5 years

A total of 213 stone formers who consulted the Department of Urology at Nagoya City University Hospital and underwent 24-hour urine collection tests between January 2023 and April 2024 were included in this study (Fig. 1). Patients with specific stone-related conditions, such as idiopathic hyperoxaluria (*n* = 2), hyperparathyroidism (*n* = 2), and renal tubular acidosis (*n* = 1), as well as those on medications such as steroids (*n* = 5) or immunosuppressive drugs (*n* = 3), were excluded from the study. The included stone formers had previously undergone stone composition by infrared spectroscopy (IR) analyses, which confirmed the presence of CaOx or CaP stones. CaP is found as basic hydroxyapatite (apatite) and calcium hydrogen phosphate dihydrate (brushite). In this study, apatite which is the most common phase in CaP stones is selected. Stone formers with struvite (*n* = 10), cystine (*n* = 13), brushite (*n* = 4), or uric acid stones (*n* = 9) were excluded from the study.

The participants were allocated into two groups: first-time stone formers (FSF, *n* = 77) and recurrent stone formers (RSF, *n* = 87). Stone recurrence was defined as the formation of new stones confirmed by computed tomography or kidney-ureter-bladder imaging, the onset of new symptoms such as pain or spontaneous stone passage, or the need for surgical intervention. Among RSF, the median follow-up period was 15 years (interquartile range [IQR], 10–22 years), the median number of recurrences was 2 (IQR, 1–3), and the median interval between stone recurrences from the initial onset of urinary stones was 6.5 years (IQR, 3.8–10.8 years).

Based on the recurrence interval data, RSF were further stratified into two subgroups: high-risk RSF, defined as those with an interval to recurrence of ≤ 6.5 years (*n* = 44), and low-risk RSF, defined as those with an interval to recurrence of > 6.5 years (*n* = 43).

### The 24-hour urine collection test and the supersaturation index measurement using PHREEQC

A 24-hour urine collection test was performed using stone formers who attended the outpatient clinic between January 2023 and January 2024. All stone formers were provided with fluid intake and dietary guidance after initial onset of stone. The patients were given a 24-hour urine measuring device (U-MATE, SB-KAWASUMI, Japan) to store urine for 24 h in room temperature and asked to promptly submit it to the outpatient clinic within 2–3 h after completion. After pH measurement, each mineral element (sodium, potassium, chloride, magnesium, phosphate, urea nitrogen, urea and creatinine) was analyzed using automated biochemical analyzer (0008α LABOSPECT, HITACHI, Japan). Furthermore, oxalate levels were assessed using acid urine storage. PHREEQC chemical species software [16, 17] was used to calculate the supersaturation index (SI) values for CaOx and CaP in the urine. CaOx included the crystal polymorphs calcium oxalate monohydrate (COM), calcium oxalate dihydrate (COD), and calcium oxalate trihydrate, while CaP included apatite and brushite. In this study, the SI of COM was defined as SI.CaOx, while the SI of apatite was defined as SI.CaP, because these were the least soluble crystalline phases at body temperature. SI of COD and Brushite, which are crystallized in urine were calculated at 4 °C.

### Cooling-induced crystallization in urine

Urine collected from 24-hour urine samples was transferred into 10-mL vials and centrifuged at 2000 rpm for 5 min. Urine sediments were then examined. In some samples, small numbers of leukocytes, erythrocytes, and crystals were observed; however, bacteria were scarcely detected. Subsequently, the samples were centrifuged at 3000 rpm for 10 min. The supernatant was collected and filtered through a 0.45-µm filter to remove impurities. After filtration, all urine sediment samples were confirmed to be free of impurities.

To determine the appropriate storage temperature, these were stored in a refrigerator under three different conditions: 4 °C, 10 °C, and 20 °C. After one day of storage, the crystallized precipitates in the vials were observed under a microscope. The amount and size of the crystals increased as the storage temperature decreased (Online Resource 1); therefore, 4 °C was selected as the cooling temperature. The precipitated crystals were then observed under an optical microscope to examine their color and morphology. The observed crystals included octahedral-shaped COD crystals (Online Resource 2a), needle-shaped and asymmetrical rod-shaped calcium hydrogen phosphate dehydrate (Brushite) crystals (Online Resource. 2b, c), bulk-shaped magnesium ammonium phosphate (MAP) crystals (Online Resource. 2 d), typical lozenge-shaped uric acid (UA) crystals (Online Resource. 2e), and amorphous CaP crystals (Online Resource. 2f).

### Raman analysis

Regarding more detailed phase identification, a Raman spectrometer (RAMAN touch; Nanophoton Japan) was used. Crystals were placed on glass slides and covered with quartz glass to suppress glass artifacts. The measurement conditions were set as follows: laser wavelength, 532 and 785 nm; laser power, 1.50 ± 0.25 mW; grating, 600 gr/mm; and wavenumber range, 600–1600 cm^−1^. To distinguish crystal phases, specific peaks associated with their chemical bonds were referenced as reported previously [18–21]. Raman spectroscopy was used to confirm the composition of each crystal type (Online Resource 3).The measurement conditions were as follows: ND filter, 50% (conditions related to laser power); grating, 2400 gr/mm; and wavenumber range, 400–1600 cm^−1^.

### Statistical analysis

Data were analyzed using the software “EZR” [22]. Continuous variables are described as mean and standard deviation or median and interquartile range, while categorical variables are presented as proportions. Comparisons between groups were performed using one-way analysis of variance and Tukey’s test for continuous variables, as well as t-tests where appropriate. Fisher’s exact test was used for categorical variables. Multivariate logistic regression was performed to identify independent predictors of stone recurrence. All tests were two-sided with a P value < 0.05 indicating statistical significance, and confidence intervals were calculated at 95%.

## Results

The background characteristics of the 77 FSF and the 87 RSF are presented in Table 1. The FSF and RSF showed no significant differences in age, body mass index, sex, or medical history (hypertension, hyperlipidemia, or diabetes mellitus). The stone-former groups also showed no significant differences in relation to the administration of citrate, presence of staghorn calculi, proportion of stone constitution (calcium oxalate and calcium phosphate). Subsequently, we examined the 24-hour urine collection test results (Table 2). Most parameters showed no significant differences in total daily urine excretion, and no significant differences were observed in the SI.CaOx values and the SI.CaP values.

**Table 1:** Demographic characteristics of stone formers (FSF vs. RSF)

	FSF *n* = 77	RSF *n* = 87	*p* value
Mean Age ± SD	59 ± 13	60 ± 13	0.561
Mean BMI ± SD	23.5 ± 5.0	24.0 ± 4.28	0.527
Sex: Male (%)	46 (59.7)	60 (69.0)	0.253
DM (%)	7 (9.1)	10 (11.5)	0.798
HL (%)	14 (18.2)	17 (19.5)	0.844
HT (%)	20 (26.0)	30 (34.5)	0.308
Administration of citric acid (%)	18 (23.3)	29 (33.3)	0.082
Staghorn calculi (%)	10 (13.0)	11 (12.6)	1.0
Proportion of stone constitution			
Calcium oxalate	86.8 ± 18.9	82.5 ± 25.7	0.232
Calcium phosphate	12.4 ± 19.4	16.1 ± 24.7	0.292

FSF: first-time stone formers, RSF: recurrent stone formers, DM: diabetes mellitus, HL: hyperlipidemia, HT: hypertension

**Table 2:** 24-Hour urine collection data of stone formers (FSF vs. RSF)

	FSF *n* = 77	RSF *n* = 87	*p* value
	Mean ± SD	Mean ±SD	
24-h urinary sodium	171.6 ± 78.6	184.7 ± 74.1	0.274
24-h urinary potassium (mmol/day)	41.5 ± 18.4	45.4 ± 18.9	0.189
24-h urinary chlorine (mmol/day)	150.9 ± 75.4	162.6 ± 68.5	0.296
24-h urinary magnesium (g/day)	0.07 ± 0.03	0.08 ± 0.03	0.581
24-h urinary phosphorus (g/day)	0.71 ± 0.28	0.73 ± 0.29	0.592
24-h urinary calcium (g/day)	0.16 ± 0.08	0.19 ± 0.10	0.129
24-h urinary creatinine (g/day)	1.22 ± 0.51	1.27 ± 0.52	0.601
24-h urinary uric acid (g/day)	0.57 ± 0.25	0.54 ± 0.23	0.518
24-h urinary urea nitrogen (g/day)	7.81 ± 2.80	8.55 ± 3.16	0.118
24-h urinary oxalate (mg/day)	29.3 ± 23.0	27.6 ± 16.1	0.587
24-h urinary urine volume (ml)	1866 ± 768	1725 ± 619	0.198
24-h urinary pH	6.4 ± 0.6	6.5 ± 0.7	0.324
24-h urinary SI.CaOx	0.91 ± 0.23	0.92 ± 0.31	0.874
24-h urinary SI.CaP	6.99 ±	7.77 ± 4.24	0.216

FSF: first-time stone formers, RSF: recurrent stone formers, SI: supersaturation index, CaOx: calcium oxalate, CaP: calcium phosphate

Next, we investigated the crystalluria induced by urine cooling in 164 all stone formers. Before cooling, crystals in urine were detected in18 (10.9%) patients, whereas after cooling, they were observed in 125 (76.2%) patients. The number of participants showing each type of crystal was as follows: before cooling urine, COD crystals 17 participants (10%) and UA crystals, 1 participant (0.6%). On the other hand, after cooling, COD crystals were observed in 109 participants (66.5%); brushite crystals in 41 participants (25%); MAP crystals in 3 participants (1.8%); UA crystals in 2 participants (1.2%); and amorphous CaP crystals in 38 participants (23.2%). COD, brushite, and amorphous CaP crystals occasionally coexisted in some urine samples. Owing to the small number of observations, the MAP and UA crystal data were excluded from the statistical analysis. Statistical analysis of the presence of each crystal type among groups (FSF vs. RSF) is summarized in Table 3. The proportions of participants showing COD crystals varied between 60% and 70% across the groups but showed no significant differences. Amorphous CaP crystals were observed in approximately 20–25% of the participants in each group, without significant differences. In contrast, brushite crystals were observed in 13 participants (16.9%) in the FSF group and 28 participants (32.2%) in the RSF group, showing significant differences (*p* = 0.03).

**Table 3:** Crystalluria data obtained after cooling urine of stone formers (FSF vs. RSF)

	FSF *n* = 77	RSF *n* = 87	*p* value
Crystalluria			
COD (%)	55 (71.4)	54 (62.1)	0.247
Amorphous CaP (%)	17 (22.1)	21 (24.1)	0.853
Brushite (%)	13 (16.9)	28 (32.2)	0.030

FSF: first-time stone formers, RSF: recurrent stone formers, COD: calcium oxalate dihydrate, CaP: calcium phosphate

**Fig. 2:**
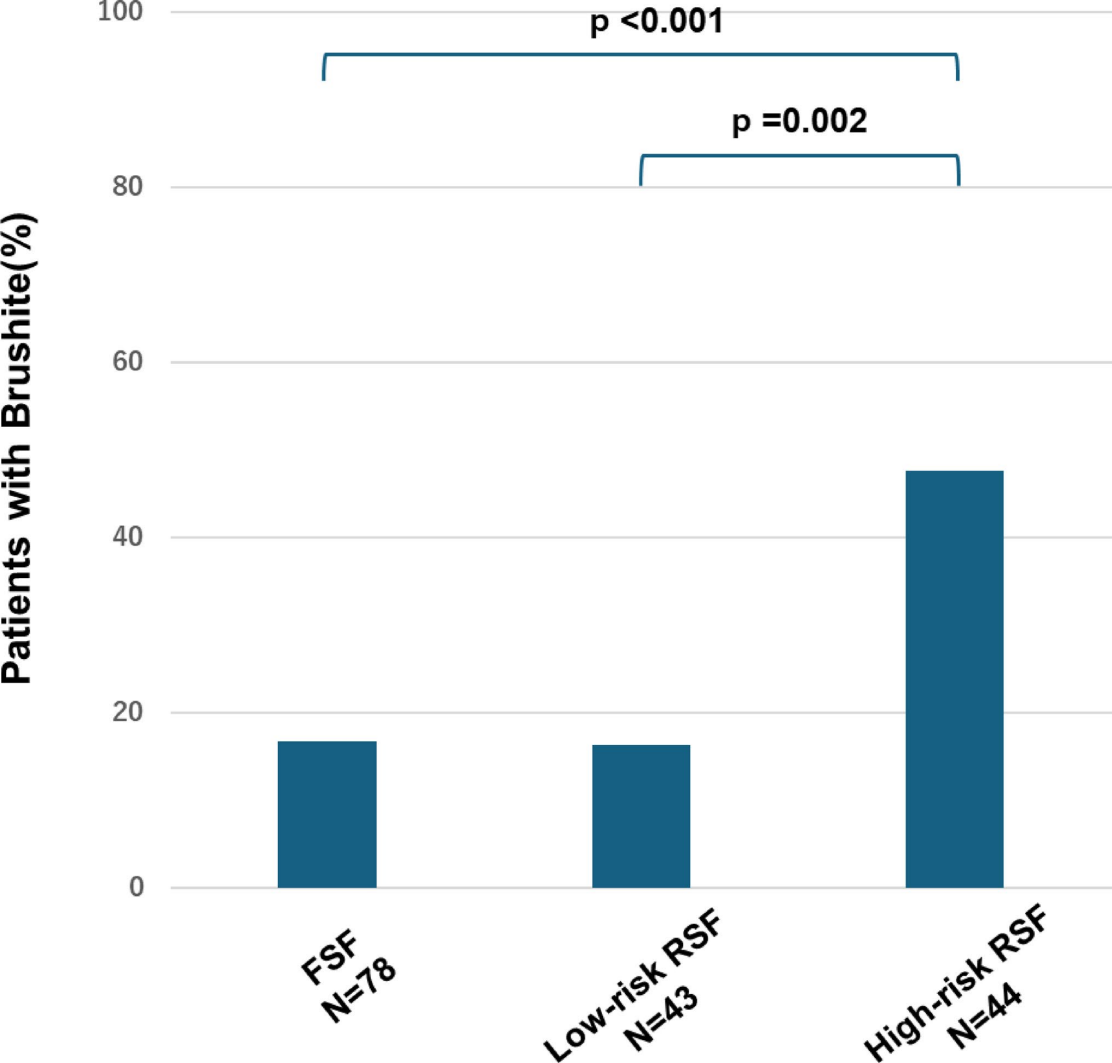
Precipitation rates of brushite crystals in cooled urine of stone formers (FSF vs. Low-risk RSF vs. High-risk RSF) compared by Tukey test FSF: first-time stone formers, RSF: recurrent stone formers

Next, we compared between two subgroups, low-risk RSF(interval to recurrence of > 6.5 years, *n* = 43) and high-risk RSF (interval to recurrence of ≤ 6.5 years, *n* = 44). There were no differences with the background characteristics or 24-hour urine collection test results. (Online Resource 4). Additionally, in the crystallization analysis of cooled urine, COD and amorphous CaP crystals showed no significant differences between the groups (Table 4). However, brushite crystals were detected in 7 low-risk RSF (16.3%) and 21 high-risk RSF (47.7%), with a significant difference (*p* = 0.002). A three-group comparison among FSF, low-risk RSF and high-risk RSF showed that brushite crystals were significantly more frequently detected in high-risk RSF (Fig. 2). Furthermore, subsequent multinomial logistic analysis indicated that the appearance of brushite crystals was the only factor showing a significant difference between the high risk RSF group and all other groups (Table 5).

**Table 4:** Crystalluria data obtained after cooling urine of stone formers (Low-risk RSF vs. High-risk RSF) FSF: first-time stone formers, RSF: recurrent stone formers, COD: calcium oxalate dihydrate, cap: calcium phosphate

	Low-risk RSF *n* = 43	High-risk RSF *n* = 44	*p* value
Crystalluria			
COD (%)	26 (60.5)	28 (63.6)	0.827
Amorphous CaP (%)	10 (23.3)	11 (25.0)	1
Brushite (%)	7 (16.3)	21 (47.7)	0.002

**Table 5:** Multinomial logistic analysis of 24-hour urine and crystalluria data for the High-risk RSF group and the other groups

	24-h urinary urine test, *p* value					Crystalluria, *p* value		
	24-h urinary calcium	24-h urinary oxalate	24-h urinary phosphorus	SI. CaOx	SI.CaP	COD	Amorphas CaP	Brushite
High-risk RSF								
Vs. FSF	0.326	0.522	0.771	0.762	0.667	0.546	0.752	0.002
vs. Low-risk RSF	0.798	0.471	0.588	0.578	0.568	0.593	0.766	0.003

FSF: first-time stone formers, RSF: recurrent stone formers, SI: supersaturation index, CaOx: calcium oxalate, CaP: calcium phosphate, COD: calcium oxalate dihydrate

**Fig. 3:**
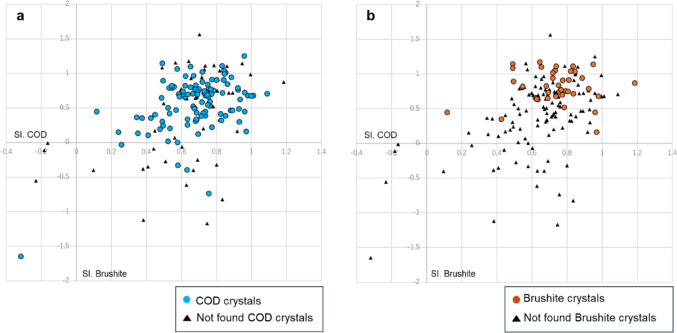
Scatter plots of crystalluria with SI.COD and SI.Brushite. (**a**) COD crystals; (**b**) brushite crystals SI: supersaturation index, COD: calcium oxalate dihydrate

To further investigate the correlation between urinary crystallization and supersaturation index, scatter plot analysis of the SI.COD and SI.Brushite values was performed of all stone formers showing COD and brushite crystals (Fig. 3). COD crystals were observed even at low SI.COD and SI.Brushite values, suggesting no correlation between COD crystallization and supersaturation index (Fig. 3a). However, SI.COD and SI.Brushite values positively correlated with brushite crystal formation, indicating that higher supersaturation levels likely contributed to brushite precipitation (Fig. 3b). Additionally, an analysis was performed to examine the relationship between urine pH and brushite crystal formation; however, no significant correlation was found between them. (Online Resource5).

**Fig. 4:**
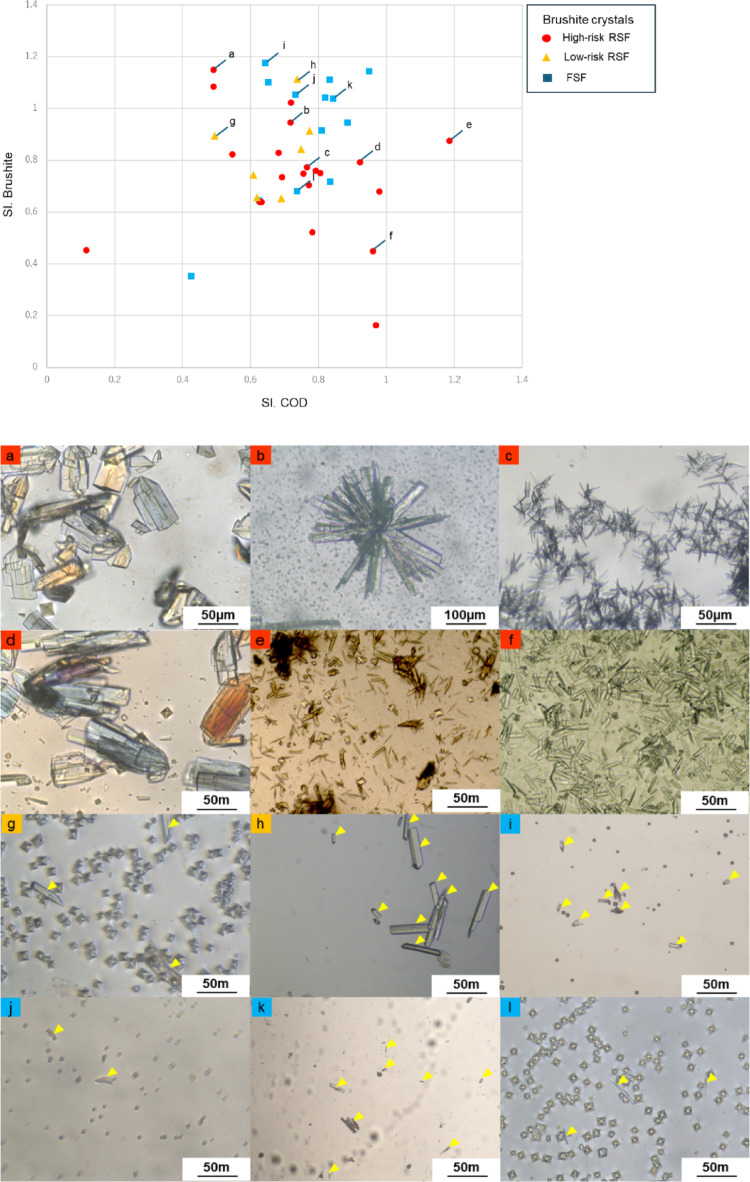
Crystalluria morphology and size of brushite crystals in the High-risk RSF, Low-risk RSF and FSF. (**a**)-(**f**) High-risk RSF groups; Almost all crystals are brushite crystals, which are large and abundant. (**g**)-(**h**) Low-risk RSF groups and (**i**)-(**l**) FSF; Brushite crystals shown with yellow arrows are small and few in amounts. The other coexisting crystals are COD crystals. RSF: recurrent stone formers. FSF: first-time stone formers. SI: supersaturation index, COD: calcium oxalate dihydrate

Furthermore, we extracted patients who exhibited brushite crystal precipitation in Fig. 4b and categorized them into FSF, low-risk RSF, and high-risk RSF (Fig. 4). Additionally, representative images of brushite crystals in urine from each patient are shown in Fig. 4a–l. In high-risk RSF patients (Fig. 4a-f), all precipitated crystals were brushite, and their quantity and size (> 100 μm) were significantly larger. In contrast, in low-risk RSF (Fig. 4g-h) and FSF (Fig. 4i-l), brushite crystals were fewer in number and smaller in size. Finally, we evaluated whether supersaturation levels differed among the three groups; however, no apparent differences were observed (Online Resource 6). These results suggest that the increased quantity and size of brushite crystals in high-risk patients cannot be solely explained by supersaturation.

To further assess the correlation between urinary brushite crystallization in cooled urine and stone composition, we analyzed the four excluded patients with brushite-based stones. Among them, three exhibited brushite crystal precipitation in cooled urine. This finding further supports the strong association between urinary brushite crystallization and stone composition, particularly in patients with a high risk of recurrence.

## Discussion

The 24-hour urine collection test can identify the risk of stone recurrence by calculating the concentration and supersaturation of mineral elements in the urine, facilitating appropriate medication and dietary guidance for stone formers [23, 24]. Previous reports have shown that risk of stone recurrence exhibit hypercalciuria, hyperoxaluria, hypocitraturia, hyperuricosuria, and hypomagnesuria [25, 26]. However, in this study comparisons among stone formers (FSF vs. low-risk RSF vs. high-risk RSF) revealed no significant differences in the 24-hour urine collection test included SI.CaOx and SI.CaP (Table 2, Online Resource4). These findings suggest that predicting recurrence risk among stone formers is not suitable. Our results are consistent with those in recent studies showing that the completion of 24-hour urine tests was not associated with a reduction in urinary stone recurrence [4]. Moreover, a review of clinical studies suggested that the 24-hour urine collection test is susceptible to the influences of daily life, leading to variability in results and further complicating the prediction of stone recurrence [5, 6].

In this study, we introduced a new method to comprehensively evaluate urine, a complex environment, by forcibly inducing crystallization in cooled urine and evaluating the differences in crystal formation. In ambient urine, crystals were detected in only 10% of samples, most of which were calcium oxalate dihydrate (COD), consistent with the detection rate reported in Reference 11. However, cooling the urine resulted in crystal detection in 80% of samples, allowing for the observation of various crystal types that were not detectable at room temperature. The observations revealed six types of crystals in stone formers (Online Resource 2). Among these crystals, COD crystals were the most commonly observed, appearing in 60–70% of stone formers (Table 3). However, the occurrence of COD crystals did not differ significantly among stone formers (FSF vs. low-risk RSF vs. high-risk RSF). Previous reports have indicated that COD crystals are frequently observed in the urine of healthy individuals and show no significant difference in comparison with stone formers, consistent with our findings [27, 28]. Therefore, COD crystal evaluations are unsuitable for predicting the risk of stone recurrence. Amorphous CaP crystals were observed in 20–25% of stone formers, and their occurrence showed no significant differences among the groups. In contrast, brushite crystals were significantly more common in high-risk RSF (47.7%) than in FSF (16.9%) and low-risk RSF (16.3%) (Fig. 2). Furthermore, multinomial logistic analysis indicated that the appearance of brushite crystals was the only factor that showed a significant difference between the high risk RSF group and all other groups (Table 5). These results suggest that the presence of brushite crystals in urine is a characteristic finding in the high-risk RSF group.　Further analysis of the quantity and size of brushite crystals in urine showed that the high-risk RSF group had more and larger brushite crystals than did the FSF and low-risk RSF groups (Fig. 4). These findings indicate that the precipitation of brushite in urine, as well as its quantity and size, are important indicators for risk of stone recurrence. Previous reports have suggested that hypercalciuria and hyperphosphaturia increase the likelihood of brushite crystallization [10.29], which is consistent with our findings (Fig. 3b). However, no correlation was found between the quantity and size of brushite crystals and the SI.COD or SI.Brushite. Previous studies have also reported that higher urine pH is an important factor in calcium phosphate crystallization [29], yet our findings suggest that urine pH alone does not contribute to brushite crystal formation (Online Resource5). These results are particularly noteworthy, as they indicate that while high supersaturation of CaOx and CaP is necessary for brushite crystal formation, crystal size and quantity are influenced by additional factors beyond supersaturation. This suggests the presence of other determinants governing brushite crystal nucleation and growth.

Urine contains many organic components, including proteins, that significantly affect stone formation [30]. Factors beyond supersaturation, such as proteins in the urine, have been suggested to play a role in crystallization. Previous in vitro studies have shown that the presence of proteins in solutions can direct precursor crystallization, inhibit pathological mineralization, and initiate mineral resorption [31, 32]. Our previous research also visualized the spatial distribution of multiple proteins and revealed the different effect of each protein on crystal growth [7]. We hypothesized that urine with a high tendency to form brushite crystals contains specific proteins that promote crystallization. Previous studies evaluated the proteins in brushite crystals precipitated from human urine and reported that the quantity and types of proteins in brushite crystals differ from those in other crystals [33, 34]. Other studies have suggested that stone formation is associated with inflammatory pathways in papillary tissue and urine, particularly brushite stone formation increased neutrophil markers in stone matrix compared with those with CaOx stones [35–38]. These findings suggest that the process of stone formation involves increased inflammation in the body, leading to higher levels of inflammatory proteins in urine, which in turn creates a conducive environment for brushite crystallization.

Brushite in stone composition is widely recognized as a risk factor for stone recurrence. However, no previous studies have reported brushite crystals in urine as a direct risk factor. One possible reason is that brushite is a metastable phase with high solubility, making its detection in ambient urine difficult. Even if brushite crystals are present in the urine, they may exist only in trace amounts or as microscopic crystals at room temperature, making them undetectable by conventional methods.

In this study, among the four excluded patients with brushite-based stones, three exhibited brushite crystal precipitation in cooled urine, indicating a strong correlation between urinary brushite crystallization and stone composition. This suggests that the presence of brushite crystallization in cooled urine may serve as an indicator of brushite crystals within kidney stones. Previous studies have shown that IR analysis alone may not detect trace mineral components within kidney stones [39]. Our previous research analyzed kidney stones which primarily were composed of CaOx and apatite, identifying RP formed by apatite crystals in thin sections of kidney stones and observing the structures where CaOx crystals had developed [9]. Additionally, we simultaneously observed microtissues where CaOx crystals had formed on brushite crystals precipitated from urine, separate from the apatite regions. Furthermore, our in vitro studies have suggested that calcium phosphate crystals may promote the nucleation and growth of calcium oxalate (CaOx) crystals, supporting the idea that brushite-like crystals in urine could facilitate calcium stone formation [40]. These significant findings suggest that even in kidney stones primarily composed of CaOx and apatite, small amounts of brushite crystals may be present and contribute to CaOx crystal formation.

Cooling the urine artificially increased supersaturation, inducing brushite crystals nucleation and growth, thereby enabling its detection. Our findings demonstrate that brushite crystallization is a distinct characteristic of high-risk recurrent stone formers. This approach provides a novel method for identifying patients at higher risk of recurrence by detecting urinary brushite crystals that would otherwise go unnoticed under normal conditions.

This study used 24-hour urine collection to examine the relationship between the average concentration of mineral components in daily urine and their crystallization. However, the 24-hour urine collection test imposes significant psychological and financial burdens on patients, limiting its frequent use in clinical practice. Early morning urine is known to have the high tendency for crystallization and correlates well with the average concentrations of substances such as Cr and Ca in 24-hour urine samples [14.26]. Therefore, future studies should focus on evaluating crystalluria in early morning urine samples as a more practical alternative. Another limitation of this study is the lack of precise quantification of crystal size and number. As shown in Fig. 4, brushite crystals tend to overlap, making accurately measuring and counting individual crystals through visual inspection alone difficult. To address this, future research incorporates image processing techniques and develop advanced analytical instruments to enable more precise quantification.

A key strength of our study is the forced precipitation of crystals by simply cooling the urine, which increased supersaturation without adding extra solutions. This method allows us to detect crystals that are typically undetectable at room temperature. In addition to the presence of brushite, the size and number of crystals are crucial factors for accurately identifying patients at risk of early recurrence. This approach offers a highly versatile, simple, and cost-effective approach for predicting urolithiasis recurrence. By improving the accuracy of recurrence prediction, it enables targeted interventions and personalized treatment plans, ultimately enhancing patient care. Furthermore, these findings highlight its potential to reduce patient suffering and alleviate the economic burden on healthcare systems.

## Supplementary Information

Below is the link to the electronic supplementary material.

Supplementary Material 1

## Data Availability

No datasets were generated or analysed during the current study.
