# Supplementary material for: Cooling-induced brushite crystallization in urine as a predictive risk marker for calcium kidney stone recurrence
Source: Urolithiasis. 2025 Sep 6;53(1):172. doi: 10.1007/s00240-025-01820-2 (PMC12414014; doi:10.1007/s00240-025-01820-2)
Supplement: Supplementary file 1 — Supplementary Material 1 [file 240_2025_1820_MOESM1_ESM.docx]

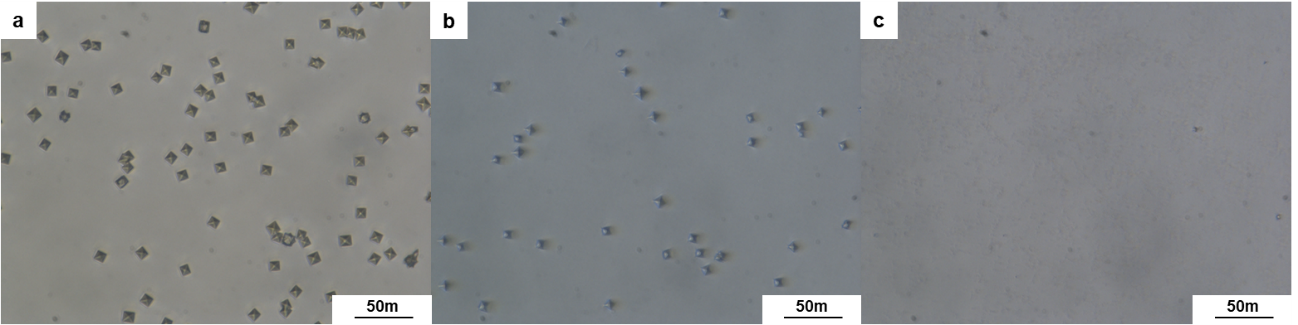


**Online Resource 1** Crystalluria under three different conditions: (a) 4°C, (b) 10°C, and (c) 20°C. The amount and size of calcium oxalate dehydrate crystals increased as the storage temperature decreased.


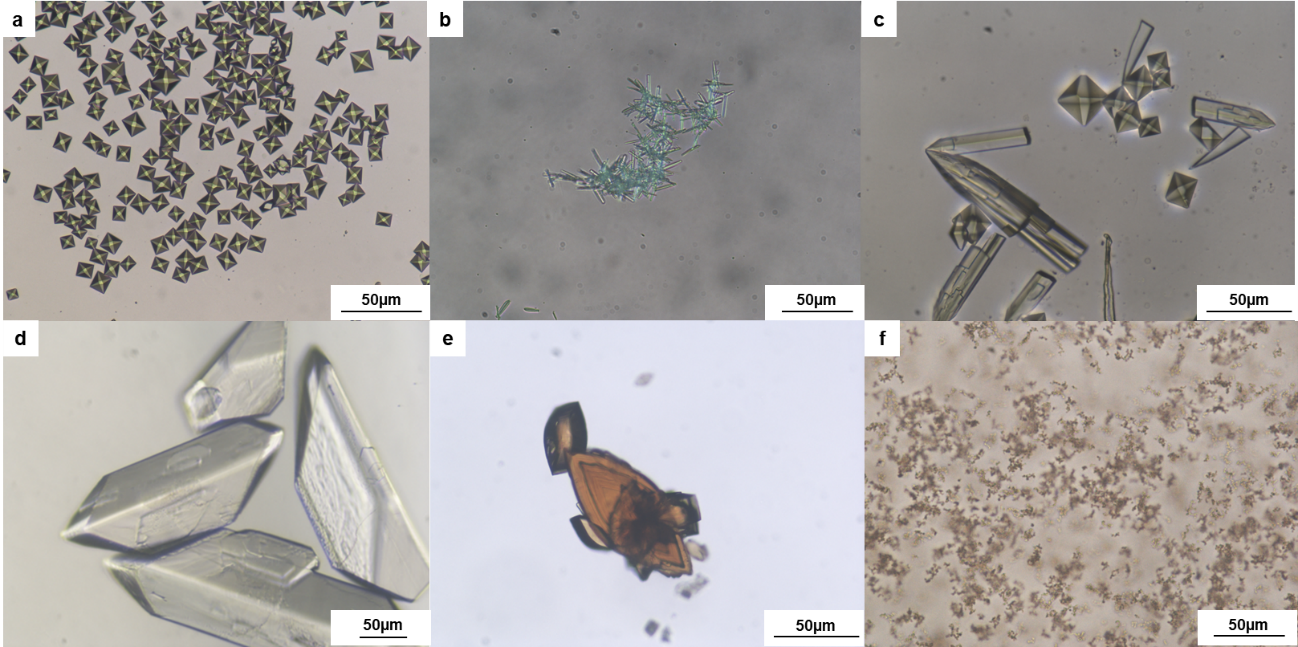


**Online Resource 2** Crystalluria induced by cooling urine. (a) Octahedral-shaped calcium oxalate dihydrate crystals, (b) needle-shaped calcium hydrogen phosphate dihydrate crystals, (c) asymmetrical rod-shaped calcium hydrogen phosphate dihydrate crystals, (d) bulk-shaped magnesium ammonium phosphate crystals, (e) typical lozenge-shaped uric acid crystals, and (f) amorphous calcium phosphate crystals

**
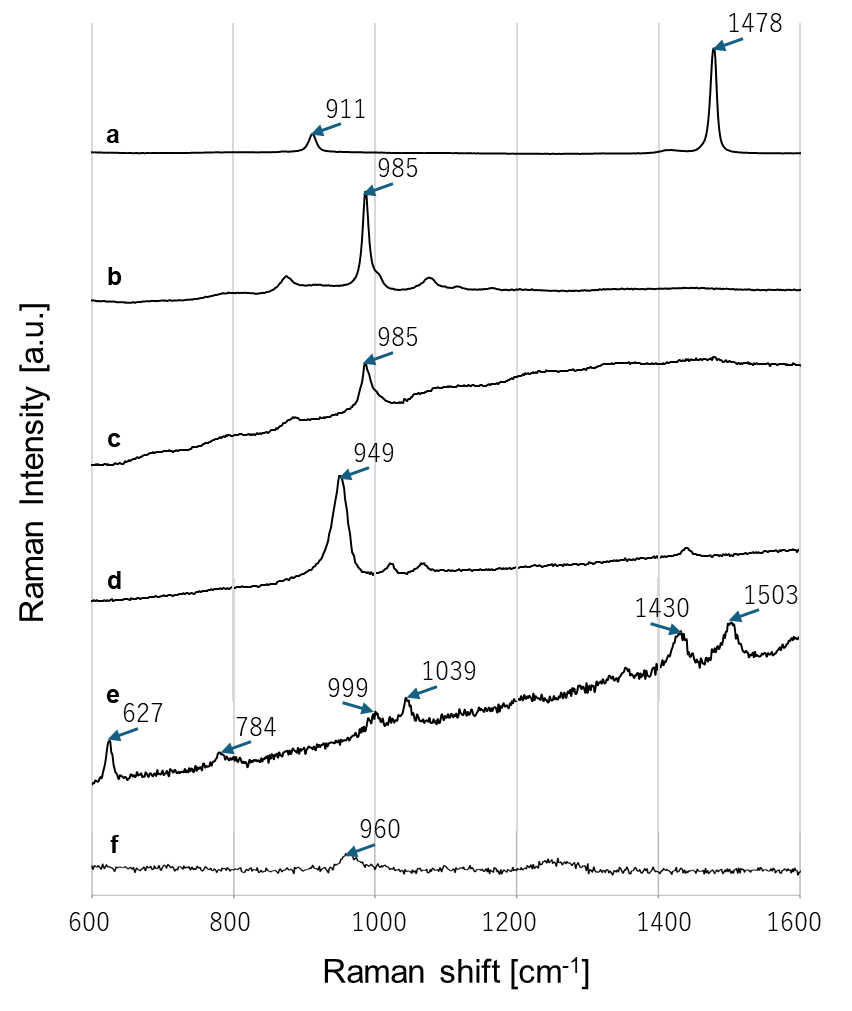
**

**Online Resource 3** Each crystal phase confirmed by Raman analysis. (a) Octahedral-shaped calcium oxalate dihydrate crystals; (b) needle-shaped calcium hydrogen phosphate dihydrate crystals; (c) asymmetrical rod-shaped calcium hydrogen phosphate dihydrate crystals; (d) bulk-shaped magnesium ammonium phosphate crystals; (e) typical lozenge-shaped uric acid crystals; and (f) amorphous calcium phosphate crystals.

|  | | **Low-risk RSF**  **n=43** | | **High-risk RSF**  **n=44** | | ***p* value** |
| --- | --- | --- | --- | --- | --- | --- |
| Mean Age ±SD |  | 60 | ±11 | 59 | ±15 | 0.642 |
| Mean BMI ±SD |  | 24.5 | ±2.9 | 23.6 | ±5.2 | 0.313 |
| Sex: Male (%) |  | 32 | (74.4) | 28 | (63.6) | 0.355 |
| DM (%) |  | 3 | (7.0) | 7 | (15.9) | 0.314 |
| HL (%) |  | 8 | (18.6) | 9 | (20.5) | 1.0 |
| HT (%) |  | 14 | (32.6) | 16 | (36.4) | 0.822 |
| Administration of citric acid (%) |  | 13 | (30.0) | 19 | (43.1) | 0.258 |
| Staghorn calculi (%) |  | 8 | (18.6) | 3 | (6.8) | 0.118 |
| Proportion of stone constitution |  |  |  |  |  |  |
| Calcium oxalate |  | 85.7 | ±21.4 | 79.3 | ±29.2 | 0.251 |
| Calcium phosphate |  | 13.5 | ±21.7 | 18.7 | ±27.4 | 0.328 |
|  |  |  |  |  |  |  |
| 24-h urinary sodium | (mmol/day) | 189.8 | ±70.8 | 179.7 | ±77.7 | 0.526 |
| 24-h urinary potassium | (mmol/day) | 45.9 | ±19.3 | 44.9 | ±18.7 | 0.810 |
| 24-h urinary chlorine | (mmol/day) | 166.4 | ±67.8 | 159.0 | ±69.7 | 0.612 |
| 24-h urinary magnesium | (g/day) | 0.08 | ±0.04 | 0.08 | ±0.03 | 0.949 |
| 24-h urinary phosphorus | (g/day) | 0.73 | ±0.26 | 0.73 | ±0.32 | 0.957 |
| 24-h urinary calcium | (g/day) | 0.18 | ±0.09 | 0.19 | ±0.11 | 0.533 |
| 24-h urinary creatinine | (g/day) | 1.28 | ±0.37 | 1.25 | ±0.64 | 0.777 |
| 24-h urinary uric acid | (g/day) | 0.50 | ±0.21 | 0.58 | ±0.25 | 0.127 |
| 24-h urinary urea nitrogen | (g/day) | 8.33 | ±2.56 | 8.76 | ±3.66 | 0.526 |
| 24-h urinary oxalate | (mg/day) | 26.8 | ±13.4 | 28.3 | ±18.6 | 0.667 |
| 24-h urinary urine volume | (ml) | 1732 | ±663 | 1718 | ±579 | 0.914 |
| 24-h urinary pH |  | 6.5 | ±0.8 | 6.5 | ±0.5 | 0.923 |
| 24-h urinary SI.CaOx |  | 0.88 | ±0.38 | 0.96 | ±0.23 | 0.255 |
| 24-h urinary SI.CaP |  | 7.53 | ±5.31 | 8.01 | ±2.86 | 0.597 |

**Online Resource 4** Demographic characteristics and 24-Hour urine collection data of stone formers (Low-risk RSF vs. High-risk RSF)

RSF: recurrent stone formers, DM: diabetes mellitus, HL: hyperlipidemia, HT: hypertension SI: supersaturation index, CaOx: calcium oxalate, CaP: calcium phosphate


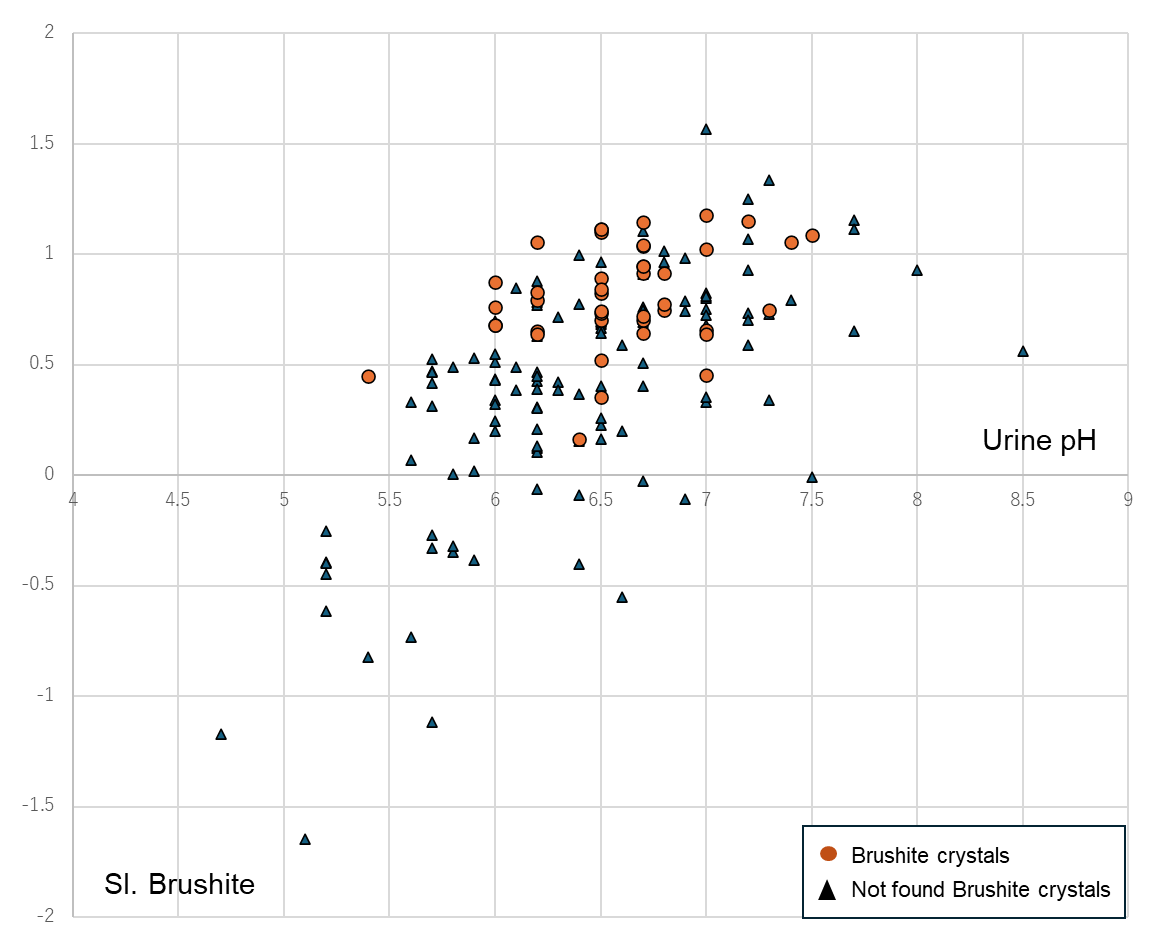


**Online Resource 5** Scatter plots of brushite crystalluria with SI.Brushite and urine pH.

SI: supersaturation index

**Online Resource 6** SI. COD and SI. Brushite among brushite crystal groups (FSF vs. Low-risk RSF vs. High-risk RSF) compared by Tukey test.

|  | **Brushite crystal groups (N=41)** | | | | | | ***p* value** |
| --- | --- | --- | --- | --- | --- | --- | --- |
|  | **FSF**  **n=13** | | **Low-risk RSF**  **n=7** | | **High-risk RSF**  **n=21** | |  |
| SI.COD | 0.75 | ±0.13 | 0.67 | ±0.10 | 0.74 | ±0.21 | 0.575 |
| SI.Brushite | 0.92 | ±0.25 | 0.83 | ±0.16 | 0.75 | ±0.22 | 0.114 |

FSF: first-time stone formers, RSF: recurrent stone formers, SI: supersaturation index, COD: calcium oxalate dihydrate.
